# Excision of Inferior Maxillary Nerve

**Published:** 1871-05

**Authors:** 


### ARTICLE IX.

### *Excision of Inferior Maxillary Nerve.*

The patient now before you has been suffering for nearly three years with a most intense neuralgia of the right side of his face and jaws. He states that he never experienced any difficulty until the three years back (some time after the close of the war, through which he passed), but that since that time he has been living such a life of pain as to render existence almost unendurable.

As to the cause of this difficulty, I regret to say that I am unable to give you a positive explanation. In a previous lecture I told you that a cause always existed, and that we could not discover it did not prove its absence, but merely our ignorance; yet such ignorance is often excusable, since this cause may be beyond present human knowledge. I have examined this man thoroughly, and have failed to find any special recognizable factor. His upper and lower denture are perfect, and after the most rigid scrutiny I am unable to find any of the nine or ten favoring conditions of neuro-odontalgia, which I then mentioned. There is no tenderness or even peculiarity of sensation afforded by any of the teeth; therefore, although these organs are so very frequently connected with this difficulty, yet in the present instance such does not appear to be the case, neither can he be suffering from the form of neuralgia resulting from the pressure upon the nerves of the alveolar process in edentulous persons from contraction and absorption, for he has all his teeth. We must, therefore, continue our investigations to other parts, and seek there an explanation by "reflex irritation."

First in order, of course, would be the other branches of this same fifth nerve; and here we find, as I have already said, no difficulty with the upper teeth; neither can we discover any tumour, wound, cicatrix or lesion upon any of the other branches which would seem to be a factor in its production.

We must then go still further yet, and examine other nerves which might reflect an irritation upon this one, and prominent among such are the branches of the cervical and brachiæ plexus, which in some cases would seem to possess a special and exceptional communication with the fifth, attributable only to a congenital or acquired peculiarity of organization. It has been found in several cases that a wound of the ulnar nerve was sufficient to cause reflex neuralgia in the fifth; and if this be true, why may not other nerves reflect their irritation in a similar manner? We have passed over and thoroughly examined every organ and portion of this man's body, reviewing every observation and fact which might enlighten us, but all has been without success, so far as the discovery of any exciting cause is concerned. We must, therefore, acknowledge the fact that we are unable to discover the special cause; yet from the expression, so to speak, of the case, I am confident that the point of irritation, though there is no tenderness in any place, is upon the inferior maxillary branch of the fifth pair, and that this point is situated somewhere in the lower jaw, anterior to the posterior dental foramen, and in the canal. Granting that such is the case, then, what could be more rational than to disconnect the point of irritation and the central point taking cognizance of such irritation—to sever the wire leading to the central office and thus prevent the transmission of any disturbing condition? To accomplish this we must make a section of the nerve at some point in its course, which is, as you know, from the ganglion of Gasser, out of the oval foramen, through the spheno-maxillary fossa, into the posterior dental foramen, through the canal and out upon the face at the mental foramen.

Now, the point of emergence from the foramen ovale, just at the optic ganglion would, perhaps, be the most effectual point; but this is deeply situated, and the dangers of the operation would be very great from the close proximity of so many important structures, the most dangerous of which would be the internal maxillary and carotid arteries. This then being impracticable, we select some point in the dental canal where it can be easily reached by simply removing the external bony palate, and in the present instance, as we wish to be certain of operating behind the irritated portion, we will go back almost to the dental foramen. Of course, such an operation should not be attempted until every means has failed, and until we are perfectly satisfied that there is no patent reason which can be otherwise removed. Again would I caution you to study well each case, and remember that over-study, venereal excess, indigestion, malaria, non-aeration of the blood, grief, tobacco, noxious gases, etc., etc., may all act as depressions of the vital economy either by over-stimulation or by under nutrition, and thus assist in the production of a continued neuralgia.

Again, moreover, all medical means should be tried to assist the removal of any lesion which may be discovered as the existing cause, yet should this be unrecognizable, we can only treat upon general principles, acknowledging our our weakness. "What then are the medical means?" you may ask, and in reply I can only recommend a few scores of drugs which have been lauded for neuralgia.

The general condition of the health should first receive our attention, since we shall often find neuralgia coexisting with anæmia, a condition which is often, in fact, the direct cause of the pains. Dr. HANDFIELD JONES has long advocated the opinion that nerve pain is invariably, and in all its phases and consequences, an expression of debility of function. In these cases, and in nearly all neuralgic cases, much benefit will be derived from the exhibition of tinct. ferri chlor. gtt. xv ad xx t. d., either alone, or better in combination with quin. sulph. gr. ij. A solid preparation of iron may be used with the same companion in pill form, with strychniæ sulph gr. 1–40 advantageously added to it.

By this building and strengthening the constitution the exciting cause, debility, may be removed and cessation of pain be the result. The anti-neuralgic remedies which have for their object the soothing and quieting of pain, either by being addressed to the system at large, or to the local affected part, are almost innumerable, and every practitioner has his favorite. A combination of quinine, conium and aconite is a most excellent anti-neuralgic, as is also potass. brom. in 20 grain doses four times a day, or zinc. valerianat, in doses of ½ to one grain t. d.

A continued course of arsenic or liq. hydr. iod. et arsen, will often be of benefit.

During the paroxysms morphia and ether are our chief reliance, together with hot mustard plasters, or hot sand or salt bags to the affected part. A broken local application of ether, by means of a sponge will often change the condition of the nerve currents, and pain will cease.

The hypodermic injection of atropiæ gr. 1–30 to 1–60, persistently continued for several weeks, is often of the most marked advantage, and the same may be said of morphia, though the latter is not so properly curative in its action.

Creasote is a good local obtunder in the strength of gtt.v to ℥j. of lard. Acenitia and veratria are also used locally in ointments, but the two articles which will probably give the greatest relief are atropia and morphia, administered subcutaneously.

Should all means fail, however, and our minds become satisfied that there exists an otherwise irremedial condition in the nerve substance, external to the proposed line of section, we are justified in performing such an operation, even though it must be confessed that the results of such interferance are not always as satisfactory as might be wished. In order to reach this nerve in the canal we must cut down through the masseter muscle, and carefully avoid all important structures. Let us look at the anatomy. Here is the facial artery, crossing the border of the jaw, just in front of the edge of this masseter muscle, and running toward the oral angle. It must not be cut, and I will fix its position by an expedient which should always be adopted by young surgeons, *i. e.*, mapping out the lines with ink. Above is the duct of Steno, extending from the lobe of the ear to the second molar tooth of the upper jaw, and we will also mark its course. Again, upon the outside I mark the outline of the parotid gland, a structure which must escape injury if possible, since salivary fistula is apt to follow.

Here, then, are three lines—the boundaries of a triangle, and having them constantly before us we can cut without fear, so long as our knife does not reach them. In the present instance we desire to go far back into the canal, and consequently we shall be obliged to divide a portion of the masseter muscle; but even this will not give us any uncontrollable hemorrhage.

The incision (which must extend down to the bone), will be somewhat semi-circular in order to allow the flap to turn back without difficulty; then, when the periosteum is divided, a trephine will be applied in order to remove this bony plate, when the cavity will be exposed and the nerve uninjured if care has been used. Then, separating the nerve from the veins and artery, a half inch or more may be removed, after which the flaps are only loosely laid in position, that healing may be facilitated while free drainage is also secured. In regard to the separation of the nerve from its accompaniments, it is sometimes possible, but even the cutting of the other structures will not produce hemorrhage sufficient to require more than a plug of lint.

[Operation then performed as described, and a portion of the nerve removed without difficulty. The hemorrhage easily controlled and simple warm water dressing used. The man had no pain for twenty-four hours, but it then returned, the paroxysms being quite frequent and severe for nearly ten days, though not so acute in character as before the operation, he being able to remain comfortable by the use of forty minims of Magendie's solution, hyperdermically per diem, while formerly he was obliged to use eighty.

This continuance was not, however, looked upon as foreshadowing the failure of the operation, since it could not be expected that a nerve so long irritated, and in such an excited condition, would regain a state of tranquility at once any more than would a rough sea for days after the storm had ceased.

Of course this delay was extremely discouraging to the patient, but at the end of two weeks the attacks slowly diminished in frequency and force, until the twinges were scarcely perceptible, and at the present time, seven weeks from the performance of the operation, he writes that he is "*entirely* free from all pain, and can use his jaws as well as ever, without exciting any discomfort."

In regard, gentlemen, to the real and permanent benefit to be derived from this serious operation I can only refer you to the literature upon the subject, and from this I feel assured that it is well worth a trial in these persistent cases, since many recoveries have undoubtedly occurred.

This inferior dental nerve is the one most easily reached, but the superior maxillary has also been several times the seat of a similar operation. In the Cincinnati *Lancet and Observer*, Aug. 1869, a case is recorded by Dr. Murray, in which the inferior was divided in the canal, and the superior as far back as the foramen rotundum, behind the ganglion of Meckel.

There are several of these excision cases reported in the *American Journal of Medical Sciences*, Jan., 1868; also one in July, 1869, in which latter case not only was the inferior nerve removed from the entire extent of the canal, but the superior also, as far back as the foramen rotundum, behind the ganglion of Meckel. This case was successful for sixteen months at least, up to the time of the report, which must have been a godsend to one who had endured untold agonies for eleven years; and I trust that a similarly happy result will occur in the patient who has just been carried from this room, since a continuance of his present condition would seem almost an impossibility, and his mind has certainly already suffered by the extreme severity of these paroxysms, which have scarcely left him five minutes of continuous sleep during the twenty-four hours.

I trust that we shall not be obliged in this case to excise the superior maxillary nerve, for that becomes a much more serious operation; still, it has been successfully done in a number of instances, and when necessary, it is best accomplished by the operation of CARNOCHAN, which consists in trephining the anterior wall of the antrum, breaking up the osseous walls of the infra-orbital canal piercing and breaking through the posterior antral wall, and cutting off the nerve just as it has emerged from the round foramen. In this operation he insists that the spheno-palatine ganglion shall be removed, since, being composed of gray or vesicular matter, it is a generator of nerve power. His operation is certainly far more simple and safe than one of LINHART, reported in the *Viertel-Jahrscrift fur die Practishe Heilkunde*, t. II., 1860, in which MIDDLEDORFF'S powerful galvano-caustic apparatus was employed. He reports that as the cauterizing current passed backward it instantaneously destroyed every structure in the spheno maxillary fossa, and an immediate gush of blood from the injured internal maxillary artery filled the orbit and all the surrounding tissues. The carotid was not tied, but hemostatic pledgets failing to arrest the hemorrhage, the actual cautery was at last applied, which is a complication no surgeon would court.

There is another case reported by NUSSBAUM, of Munich, in Gurer's "Progress of Surgery," 1863-65, Berlin, which will certainly carry off the palm, if such it be, for "heroic" treatment. This woman submitted to various sections of the supra and infra-orbital nerves, for a period of five years, "but finding no relief," the report continues, "repeated extirpations of the cicatrices were made, the common carotid tied, the ascending ramus of the lower jaw trephined, and the inferior dental nerve exsected together with the mylohyoid and lingualis, causing necrosis of the bone, which had to be removed to the articulation. Five months later the neuralgia returned, when the infra-orbital nerve was exsected nearly to the foramen rotundum. This was followed by an osteo-plastic resection of the upper part of the sup. max. bone, but saving the alveolar process, as in Langenbeck's operation. The bones were then replaced and united by first intention. The pain had entirely ceased up to the time of publication, several months after the operation.

These cases are certainly formidable, but our own American reports are, perhaps, the most favorable ones published, and certainly, lead us to hope for many favorable results, especially when we read other opinions, as referred to in the above reports from "Brauns' Surgery," Tubingen, 1859, under "Kau and Geschmachs Organs," and p. 838, erst band. Carmichael calls attention to the fact that the nerves are often enlarged and thickened in these cases of inveterate neuralgia, and a case is recorded in my book upon Oral Surgery, p. 442, where the antral nerves were of the size of knitting needles, and were removed from the antrum.

In conclusion, then, gentleman, let me sum up by saying that although, as remarked by Dr. Anstie, the subject is "an uninviting one," yet that it is an operation eminently proper, when all other means have failed, and that it promises a reasonable hope of success.—*Prof. Garretson's Clinic, in University of Pennsylvania. Reported by Dr. Willard, in Medical and Surgical Reporter.*

